# Evaluating the value of robotic versus open pancreaticoduodenectomy: improved clinical outcomes without compromising hospital revenue per admission

**DOI:** 10.1007/s00595-026-03451-5

**Published:** 2026-08-06

**Authors:** Yusuke Seki, Yoshikuni Kawaguchi, Takeshi Kano, Kyoji Ito, Satoru Abe, Yujiro Nishioka, Akihiko Ichida, Takeshi Takamoto, Nobuhisa Akamatsu, Kiyoshi Hasegawa

**Affiliations:** Hepato-Biliary-Pancreatic Surgery Division and Artificial Organ and Transplantation Division, Department of Surgery, Graduate School of Medicine, The University of Tokyo, 7-3-1, Hongo, Bunkyo-ku, Tokyo, 113- 0033 Japan

**Keywords:** Cost analysis, Open pancreaticoduodenectomy, Pancreatic fistula, Robotic pancreaticoduodenectomy

## Abstract

**Purpose:**

Robotic pancreaticoduodenectomy has been increasingly adopted; however, its economic feasibility under the Japanese national health insurance system remains unclear. This study aimed to evaluate the clinical and economic outcomes of robotic pancreaticoduodenectomy (RPD) compared with open pancreaticoduodenectomy (OPD).

**Methods:**

Patients who underwent pancreaticoduodenectomy between January 2020 and December 2024 at The University of Tokyo were identified from an institutional database. Patients with pancreatic ductal adenocarcinoma were excluded because portal vein resection and reconstruction performed on a robotic platform were not covered by the Japanese National Health Insurance System during the study period. The clinical outcomes and economic indicators, including hospital revenue, costs, and profit, were assessed under the Japanese lump-sum payment system.

**Results:**

Eighty-three patients were included in the study (41 in the robotic group and 42 in the open group). The rate of clinically relevant pancreatic fistula was significantly lower (2.4% vs. 45.2%, *P* < 0.001), and the median hospital stay was significantly shorter (7.0 vs. 22.5 days, *P* < 0.001) in the robotic group. The mean hospital revenue per admission (22,007 vs. 19,701 USD, *P* = 0.015) and profit per day (2,552 vs. 793 USD, *P* < 0.001) were significantly higher in the robotic group.

**Conclusions:**

Robotic pancreaticoduodenectomy yielded superior perioperative outcomes and shorter hospitalization than open pancreaticoduodenectomy in selected patients without compromising hospital revenue per admission.

**Supplementary Information:**

The online version contains supplementary material available at https://doi.org/10.1007/s00595-026-03451-5.

## Introduction

With advances in robotic surgery technology, the application of robotic pancreaticoduodenectomy (RPD) has been increasingly expanded worldwide as a minimally invasive alternative to open pancreaticoduodenectomy (OPD). Previous studies have demonstrated that RPD is associated with comparable safety, fewer postoperative complications, and shorter hospital stays compared with OPD [1–5].

However, the introduction of robotic surgical systems has raised concerns regarding their substantial financial implications for hospitals and healthcare systems. Reported medical costs for RPD vary considerably across countries [6–10], largely reflecting differences in healthcare structures. Some studies have shown that hospital costs per admission are similar between RPD and OPD [8, 10], , whereas others have reported that RPD is associated with reduced overall hospital costs owing to improved short-term outcomes [6, 9]. Notably, these economic evaluations have been conducted in healthcare settings outside Japan, such as in Germany and the United States. One study showed that hospital revenue per admission was higher in patients undergoing RPD than in those undergoing OPD in Japan [11]. However, no study has comprehensively evaluated the economic impact of RPD, including hospital revenue, treatment-related costs, and profit margins, within the framework of the Japanese national health insurance system. Because healthcare systems and resource allocation differ across countries, the direct extrapolation of international cost data remains challenging.

We hypothesized that the higher total costs of RPD would be offset by reduced postoperative care costs, resulting in favorable economic outcomes compared with OPD.

Therefore, this study aimed to evaluate the clinical outcomes and economic performance of RPD relative to that of OPD. Specifically, we analyzed key hospital management indicators, including the reimbursement structure, treatment costs, daily hospital profit, and operating margin, in the Japanese Health Insurance System.

## Methods

### Study population

A prospectively maintained database was reviewed to identify patients who underwent pancreaticoduodenectomy (PD) at The University of Tokyo between January 2020 and December 2024. Patients who underwent PD for pancreatic ductal adenocarcinoma were excluded because this condition often requires portal vein resection and reconstruction, a procedure not covered by the Japanese National Health Insurance System through robotic or laparoscopic platforms as of June 2026. RPD was initiated by a single surgeon in January 2023, who performed all the RPD procedures during the study period. Four patients who underwent RPD before 2022 by another surgeon were excluded to avoid heterogeneity in the economic evaluation because the devices, techniques, and perioperative management used before 2023 differed from those used thereafter. To minimize the potential selection bias between RPD and OPD during the overlapping period (2023–2024), patients who underwent OPD after 2023 were excluded. Consequently, the final analysis compared patients who underwent OPD between 2020 and 2022 with those who underwent RPD between 2023 and 2024. This study was approved by the Research Ethics Committee/Institutional Review Board of The University of Tokyo (approval number: 2158-11) and was conducted in accordance with the principles outlined in the Declaration of Helsinki. Informed consent was obtained using an opt-out approach on the institution’s website.

### Surgical procedures for pancreaticoduodenectomy

The details of the surgical procedures for OPD have been described elsewhere [5, 12] and did not change substantially during the study. The superior mesenteric artery (SMA)-first approach was used for patients with tumor extension toward the SMA. Pancreatic parenchymal transection was performed using the clamp-crushing method with ligation of the pancreatic vessels. Pancreatojejunostomy was performed using modified Blumgart anastomosis, and gastrojejunostomy was performed using a 60-mm linear stapler.

The da Vinci^®^ Surgical System (Intuitive Surgical, Inc., Sunnyvale, CA, USA) was used for RPD. The resection phase of RPD was standardized using the pancreatic head clockwise devascularization technique, which systematically devascularizes the pancreatic head in three sequential steps: division of (1) the gastroduodenal artery, (2) the first jejunal vein branches, including the inferior pancreatoduodenal vein from the pancreatic head, and (3) the inferior pancreatoduodenal artery and first jejunal artery (1st JA) [13, 14]. Pancreatojejunostomy was performed using a modified Blumgart anastomosis, and gastrojejunostomy was created with a 60-mm linear stapler through the umbilical wound in an open setting after undocking the robot [15].

### Postoperative management

Postoperative management after PD has been described elsewhere [5, 13] and it did not change substantially during the study period. For patients undergoing OPD, an enteral feeding tube was generally placed during surgery in patients undergoing OPD. Enteral nutrition was initiated on POD 1, and oral intake was initiated on POD 2. The drainage tubes were removed after confirming the absence of bile leakage or POPF. In patients with BL POPF, the drainage tubes were routinely exchanged for new tubes under radiographic fluoroscopic guidance on approximately POD 7. If the fistula was localized, the remaining drainage tube was cut short and left inserted, and the tube was gradually removed after confirming non-infectious drain output and the absence of inflammatory findings on blood examination.

For patients undergoing RPD, the institutional Enhanced Recovery After Surgery protocol, named Fast-Track Return to Normal Activity (FASTOL) program, was implemented to maximize the value of the minimally invasive approach [13]. Oral intake and mobilization exercises were initiated on postoperative day 1. The drainage tube placed at the hepaticojejunostomy site was removed on postoperative day (POD) 1 unless the patient had bile leaks, and the drainage tube placed at the PJ site was removed before POD 5 if the patient did not have an elevated level of amylase discharge from the drainage tubes. If patients had grade BL POPF based on the ISGPS definition, one of the two drainage tubes placed at the PJ was cut short and left to be inserted, and the patient was subsequently discharged. The criteria for hospital discharge were as follows: absence of postoperative complications, discontinuation of intravenous analgesia, and the ability to independently perform the activities of daily living.

### Calculation of hospital charges and surgical costs in Japan

Hospital revenue per admission was calculated using the Japanese lump-sum payment system based on the Diagnosis Procedure Combination (DPC)/Per-Diem Payment System, which corresponds to the Diagnosis-Related Groups/Prospective Payment System used in the United States [16]. Briefly, this payment system comprises a DPC component, including the hospital basic fee and charges for medications and examinations, and a fee-for-service component, which includes charges for surgical procedures and anesthesia. Under the Japanese National Health Insurance System, the surgical fee is set at USD 6,940 (972,300 yen) for OPD and USD 12,400 (1,736,400 yen) for RPD, respectively. The national reimbursement system, including surgical fees, is revised every two years if deemed necessary. The daily hospital basic fee within the lump-sum payment decreases gradually across three levels, depending on the time elapsed after hospital admission [16]. In general, 70–90% of the hospital charge is covered by the Japanese National Health Insurance System, with the exact percentage depending mainly on patient age.

Hospital costs per admission were calculated as the sum of expenses related to devices, instruments, robotic arms, and medications that were not reimbursed under the DPC lump-sum payment system. The rough balance was defined as the hospital revenue per admission minus treatment-related hospital costs per admission. Hospital profit per day was obtained by dividing the hospital profit by the length of postoperative hospital stay, and the hospital operating margin was calculated as the hospital profit divided by hospital revenue per admission. The intraoperative surgeon labor cost was estimated as the operation time multiplied by the hypothetical hourly wage of physicians in national university hospitals in Japan, as defined by the Hospital Management and Accounting System [17]: for example, the hourly wage assumption, 7,370 yen/hour for a professor; 6,281 yen/hour for an associate professor; 5,464 yen/hour for an assistant professor; 2,844 yen/hour for a senior resident/fellow; and for a junior resident, 2,427 yen/hour. This estimation was necessary because surgeon-specific labor expenses are not reimbursed by the Japanese healthcare system. Regarding the number of surgical assistants, RPD is typically performed with one assistant, whereas OPD requires two to three. The daily depreciation expense of the robotic system was estimated based on the purchase price of the robotic platform and its expected operational lifespan. The economic analysis, which included the two estimated expenses described above, was presented as a supplementary evaluation because the surgeon labor and robotic depreciation costs were not explicitly defined within the national system.

### Definition

The American Society of Anesthesiologists physical status classification system [18] was used to evaluate the patients’ preoperative physical condition, which was recorded based on the anesthesiologists’ preoperative assessments. Postoperative complications within 90 days after surgery were graded according to the Clavien–Dindo classification [19, 20]. Postoperative pancreatic fistula (POPF) was defined and categorized according to the 2016 International Study Group of Pancreatic Surgery criteria [21]. Grades B and C were considered clinically relevant postoperative pancreatic fistula (CR-POPF). Delayed gastric emptying (DGE) was defined according to the criteria of the International Study Group of Pancreatic Surgery [22]. Postoperative length of hospital stay was measured from the date of surgery to the date of discharge.

### Statistical analysis

Continuous variables related to economic outcomes were expressed as the mean ± standard deviation (SD), whereas other continuous variables were presented as the median with an interquartile range (IQR). Differences in continuous variables between the groups were analyzed using the Wilcoxon rank-sum test. Categorical variables are expressed as numbers (percentages) and were compared using Fisher’s exact test. All statistical tests were two-sided, and a P value of < 0.05 was considered statistically significant. Statistical analyses were performed using the JMP software program (version 19; SAS Institute Inc., Cary, NC, USA).

## Results

### Patient demographics and clinical characteristics

**Fig. 1:**
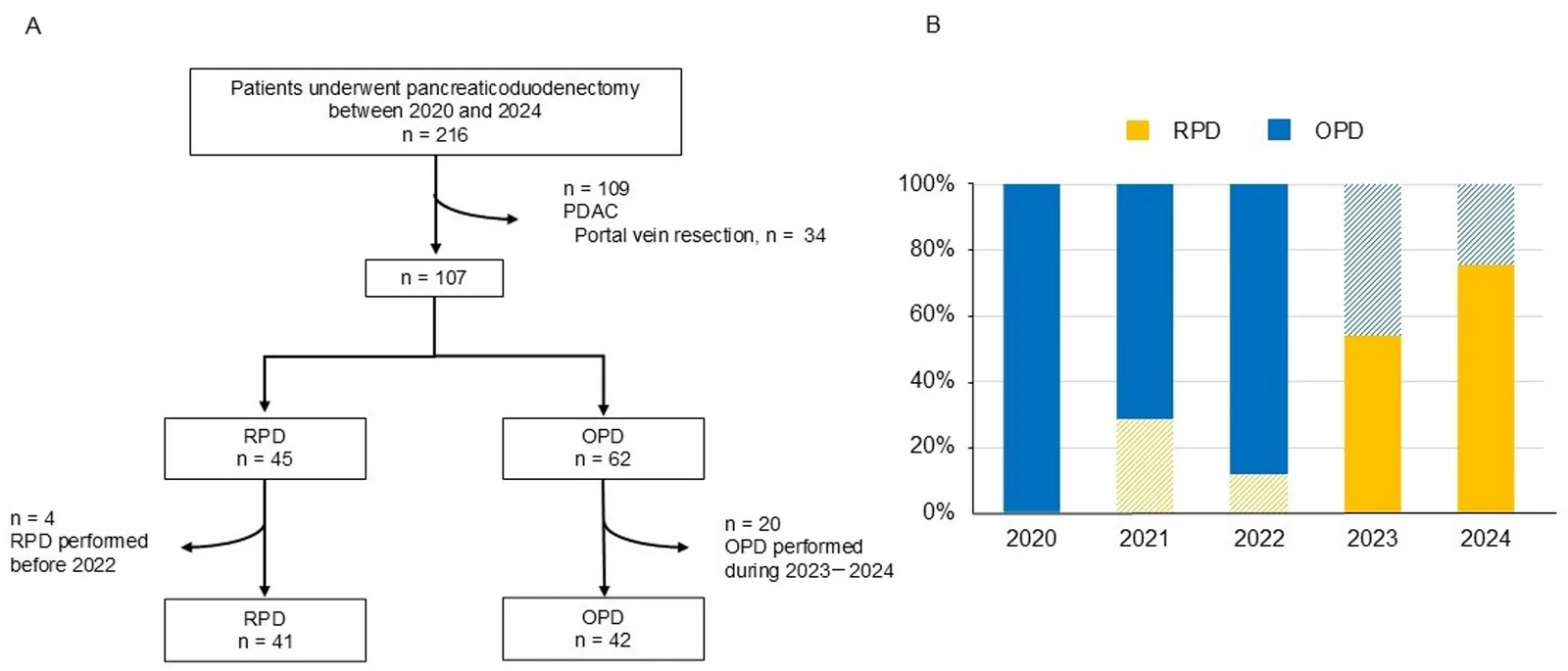
Study flow diagram illustrating patient selection (**A**) and the annual distribution of RPD and OPD (**B**). The hatched bars in panel B represent excluded cases (RPD performed before 2022 and OPD performed during 2023–2024). Abbreviations: PDAC, pancreatic ductal adenocarcinoma; RPD, robotic pancreaticoduodenectomy; OPD, open pancreaticoduodenectomy

Of the 216 patients who underwent PD, 83 met the inclusion criteria, comprising 41 who underwent RPD ( RPD group) and 42 who underwent OPD ( OPD group) (Fig. 1A). The annual distributions of RPD and OPD cases are shown in Fig. 1B. The demographic and clinical characteristics did not differ significantly between the two groups, except for the diagnosis of periampullary lesions (Table 1). The two major indications were intraductal papillary mucinous neoplasm (IPMN) and distal bile duct cancer.

**Table 1:** Comparison of Demographic and Clinical Characteristics Between Robotic and Open Pancreaticoduodenectomy

Characteristics	RPD group (*n* = 41)	OPD group (*n* = 42)	*P* value
Demographic and clinicopathological variables			
Age, median (IQR), years	68.0 (57.0–75.0)	71.0 (59.8–78.8)	0.392
Sex, male : female, n	22 : 19	30 : 12	0.115
BMI, median (IQR), kg/m^2^	22.3 (19.3–24.6)	22.7 (20.9–24.6)	0.613
ASA score ≥ 3, n (%)	9 (22.0)	7 (16.7)	0.588
History of upper abdominal surgery, n (%)	0	2 (4.8)^†^	0.494
Diagnosis, n (%)			< 0.001
IPMN	21 (51.2)	21 (50.0)	
Distal bile duct cancer^*^	10 (24.3)	17 (40.5)	
Duodenal tumor	4 (9.8)	4 (9.5)	
NEN	6 (14.6)	0 (0.0)	
Others	0 (0.0)	1 (2.4)	

Abbreviations: RPD, robotic pancreaticoduodenectomy; OPD, open pancreaticoduodenectomy; IQR, interquartile range; IPMN, intraductal papillary mucinous neoplasm; NEN, neuroendocrine neoplasm

^*^Including ampulla of Vater tumor

^†^Cholecystectomy (*n* = 1) and repair of duodenal perforation repair (*n* = 1)

### Surgical and postoperative outcomes

Table 2 summarizes the surgical and post-operative outcomes. The operation time was significantly longer, whereas the estimated blood loss was significantly lower in the RPD group than in the OPD group. The median (IQR) operation time was 683 min (620–789 min) versus 467 min (405–562 min) (*P* < 0.001), and the median (IQR) estimated blood loss was 270 mL (50–500 mL) versus 545 mL (340–1,213 mL) (*P* < 0.001). CR-POPF and DGE were significantly lower in the RPD group than in the OPD group (2.4% vs. 45.2%, *P* < 0.001; 0% vs. 19.0%, *P* = 0.005, respectively). The median (IQR) length of hospital stay was also significantly shorter in the RPD group than in the OPD group: 7.0 (5.0–9.0) versus 22.5 (17.3–35.8) days, *P* < 0.001.

**Table 2:** Comparison of Surgical and Postoperative Outcomes Between Robotic and Open Pancreaticoduodenectomy

Characteristics	RPD group (*n* = 41)	OPD group (*n* = 42)	*P* value
Operation time, median (IQR), min	683 (620–789)	467 (405–562)	< 0.001
Estimated blood loss, median (IQR), mL	270 (50–500)	545 (340–1213)	< 0.001
Complications ≥ Clavien–Dindo Grade III, n (%)	2 (4.6)	8 (19.0)	> 0.999
CR-POPF, n (%)	1 (2.4)	19 (45.2)	< 0.001
DGE, n (%)	0	8 (19.0)	0.005
Postoperative bleeding, n (%)	0	2 (4.8)	0.494
Hospital stay, median (IQR), days	7.0 (5.0–9.0)	22.5 (17.3–35.8)	< 0.001

Abbreviations: RPD, robotic pancreaticoduodenectomy; OPD, open pancreaticoduodenectomy; IQR, interquartile range; CR-POPF, clinically relevant postoperative pancreatic fistula; DGE, delayed gastric emptying

### Economic outcomes between robotic and open Pancreaticoduodenectomy

**Fig. 2:**
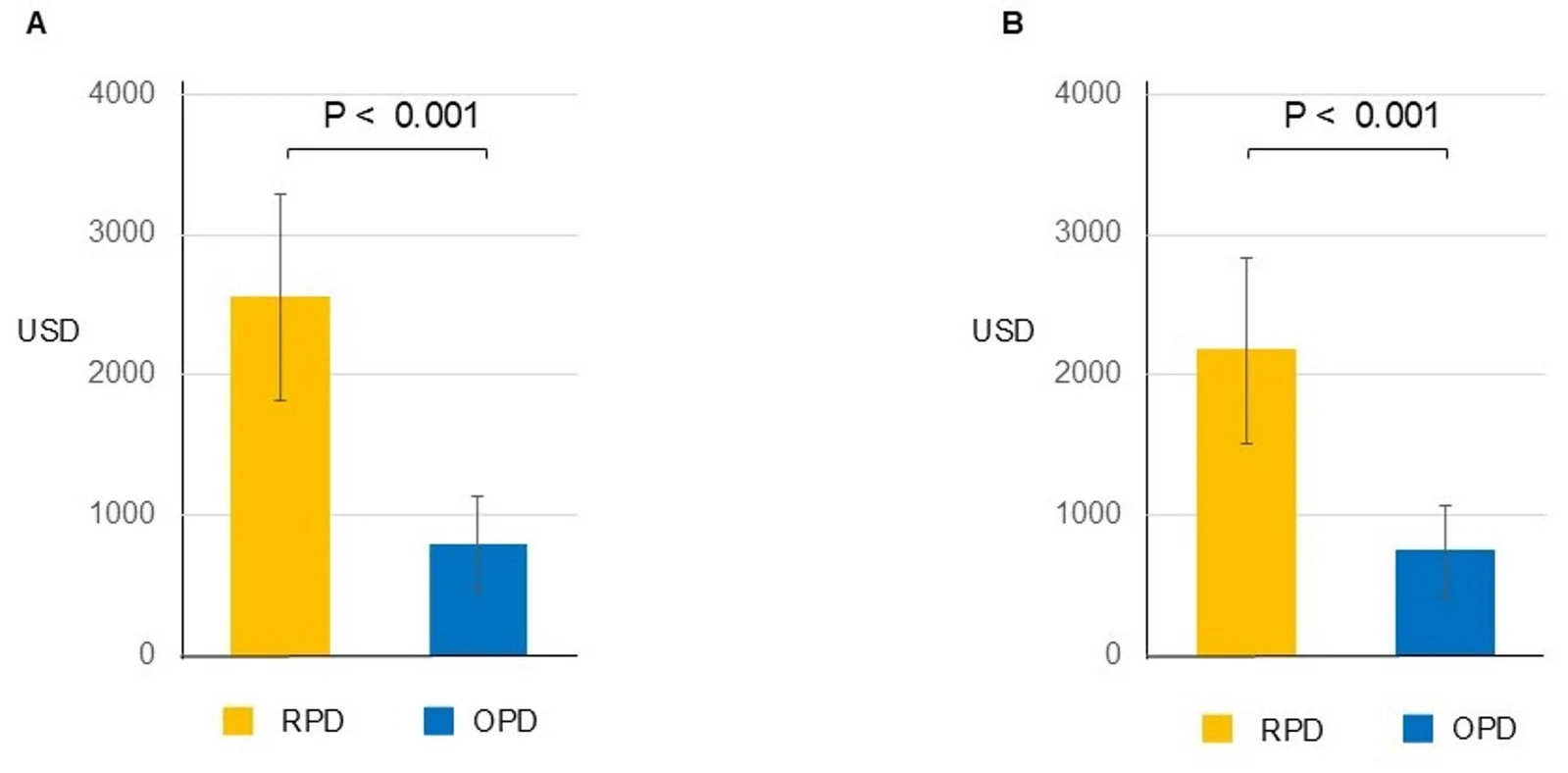
Hospital profit per day (**A**) and hospital profit per day, including estimated intraoperative surgeon labor costs and estimated depreciation costs of the robotic platform (**B**). The mean ± SD hospital profit per day was significantly higher in the RPD group than in the OPD group. (A) 2,552 ± 733 USD vs. 794 ± 342 USD, *P* < 0.001 (B) 2,174 ± 661 USD vs. 745 ± 325 USD, *P* < 0.001

Table 3 summarizes the economic outcomes of the two groups. The hospital revenue per admission and hospital cost per admission were significantly higher in the RPD group than in the OPD group (22,007 ± 1,880 USD vs. 19,701 ± 4,371 USD, *P* < 0.001; 4,712 ± 1,835 USD vs. 2,752 ± 1,827 USD, *P* < 0.001). The hospital profit did not differ significantly between the RPD and OPD groups (17,295 ± 2,399 USD vs. 16,948 ± 4,036 USD, *P* = 0.120). The hospital profit per day was significantly higher in the RPD group than in the OPD group (2,552 ± 733 vs. 793 ± 344 USD, *P* < 0.001) (Fig. 2A). In contrast, the median hospital operating margin was significantly lower in the RPD group than in the OPD group (0.79 [0.75–0.82] vs. 0.86 [0.84–0.88], *P* < 0.001).

**Table 3:** Economic Outcomes Between Robotic and Open Pancreaticoduodenectomy

Characteristics	RPD group (*n* = 41)	OPD group (*n* = 42)	*P* value
Hospital revenue per admission, mean ± SD			< 0.001
Yen	3,081,109 ± 263,255	2,758,166 ± 611,937	
USD^*^	22,007 ± 1,880	19,701 ± 4,371	
Hospital costs per admission, mean ± SD			< 0.001
Yen	659,713± 256,944	385,339 ± 115,906	
USD^*^	4,712 ± 1,835	2,752 ± 827	
Hospital profit, mean ± SD			0.120
Yen	2,421,396 ± 335,880	2,372,826 ± 565,103	
USD^*^	17,295 ± 2,399	16,948 ± 4,036	
Hospital operating margin A, median (IQR)	0.79 (0.75–0.82)	0.86 (0.84–0.88)	< 0.001

Abbreviations: RPD, robotic pancreaticoduodenectomy; OPD, open pancreaticoduodenectomy; SD, standard deviation; IQR, interquartile range

*Calculated based on an exchange rate of 140 yen per U.S. dollar (average from 2022 to 2024)

Supplementary Table 1 presents the additional economic evaluations, which included estimated intraoperative surgeon labor costs and daily depreciation expenses for the robotic system (estimated at 1,667 USD [233,318 yen] per day) as part of the hospital cost per admission. The depreciation expenses of the robotic systems were estimated based on the robot system price and expected working duration. After incorporating these expenses, the median hospital operating margin remained significantly lower in the RPD group than in the OPD group (0.71 vs. 0.80, *P* < 0.001), whereas the rough balance per day remained significantly higher in the RPD group (2,174 ± 661 vs. 745 ± 325 USD, *P* < 0.001) (Fig. 2B).

## Discussion

The present study comprehensively evaluated the institutional economic impact of RPD under the Japanese National Health Insurance System. The hospital revenue per admission was significantly higher in the RPD group than in the OPD group, although the total treatment costs were also significantly higher in the RPD group. Consequently, the overall rough balance did not differ significantly between the groups. Because patients who underwent RPD experienced markedly lower rates of CR-POPF and DGE, as well as a notably shorter hospital stay, the rough balance per day was significantly higher in the RPD group than that in the OPD group. These findings suggest that although RPD involves higher upfront costs, these expenses are offset by reduced complication rates and shorter hospitalizations, thereby offering superior economic efficiency on a per-day basis for hospitals.

The reported medical costs for RPD have varied considerably in previous studies [6–10]. Several investigations have demonstrated no significant difference in hospital costs per admission between RPD and OPD, despite the higher intraoperative expenses associated with RPD [8, 10]. Conversely, other reports have indicated that RPD is associated with a reduced length of stay and lower overall hospital costs compared with conventional management, particularly when enhanced recovery pathways were implemented or when routine postoperative intensive care unit admission was omitted [6, 9]. These discrepancies likely arise from heterogeneity in national healthcare systems, institutional cost structures, inclusion or exclusion of capital expenditures, and differences in patient selection criteria. Consequently, cross-country cost comparisons are inherently complex. Factors such as reimbursement models, hospital accounting methods, labor costs, and pricing of surgical instruments can markedly affect reported expenditures. Furthermore, variations in healthcare infrastructure and resource allocation further complicate direct international economic comparisons. Within this context, the present study evaluated the economic outcomes of RPD and OPD within the framework of the Japanese National Health Insurance System.

Robotic surgery has traditionally been regarded as a cost-intensive approach that may strain hospital financial performance owing to substantial capital investment and ongoing maintenance expenses [6, 10]. However, the present study demonstrated that RPD can have a favorable economic impact. Specifically, the mean daily hospital profit for patients who underwent RPD was considerably higher than that for patients who underwent OPD (Fig. 1A). This finding is consistent with the results of our supplementary economic assessment, which included estimated intraoperative surgeon labor costs and depreciation expenses of the robotic system (Fig. 1B). When surgical wards maintain a high bed occupancy rate (approximately 90% at our institution), improved daily financial balances indicate that RPD enhances cost-effectiveness per day and contributes positively to overall hospital economics. Although RPD generally involves longer operative times, the associated increase in surgeon labor costs was offset by the reduced need for assistant surgeons, thereby improving the overall labor efficiency within the surgical team. Despite the higher initial investment and ongoing maintenance costs, RPD appears to improve the clinical outcomes while maintaining comparable out-of-pocket expenses for patients. Furthermore, RPD may offer meaningful economic advantages to hospitals, thereby contributing positively to their overall financial performance. Increasing experiences with robotic surgery, together with technological advancements, are expected to improve the economic efficiency of RPD by further reducing the hospital costs required for operating rooms, robot platforms, and estimated labor costs.

This study has certain limitations. First, this was a retrospective, single-institution study with a relatively small sample size, which may limit the generalizability of the findings. Further studies with larger cohorts and multicenter designs are necessary to validate and extend our findings. Second, although potential selection bias between the RPD and OPD groups was a concern, this issue was addressed by excluding patients who underwent OPD after 2023, when RPD became the predominant approach for most indications. Consequently, the final analysis compared consecutive OPD cases from 2020 to 2022 with consecutive RPD cases from 2023 to 2024, thereby minimizing the overlap in the surgical selection periods (Fig. 1B). As such, the OPD group in our study is expected to represent patients who were not considered candidates for robotic surgery at the time but might have been eligible if an RPD program had already been established. Our study design may have inherently included some historical bias. However, we believe that this bias is minimal because the perioperative management and institutional learning curves for OPD did not change substantially over the past 5 years (up to 2024). The exclusion of patients with pancreatic ductal adenocarcinoma limits the generalizability of our findings. Nonetheless, this decision is counterbalanced because it avoids confounding the economic evaluation of RPD, particularly in cases requiring RPD with portal vein reconstruction, which is not covered by the national insurance system. Third, economic evaluation is inevitably influenced by institutional cost structures. To provide a more realistic estimation of economic outcomes, we performed a supplemental analysis incorporating the estimated intraoperative surgeon labor costs and depreciation expenses of the robotic system. Additionally, biennial revisions to the national reimbursement system may have influenced the current findings. In particular, a special premium for securing surgical care and robot-assisted surgery was added for institutions meeting the requirements in June 2026. Nonetheless, in the context of comparison between RPD and OPD, the influence of this revision may be limited for our study because the former premium is added for both procedures, and because the value of 150,000 yen will be simply added to the hospital revenue of the RPD group in our study.

In conclusion, RPD was associated with improved perioperative outcomes compared with OPD while maintaining patient out-of-pocket expenses comparable to those of OPD. In appropriately selected patients, RPD offers clinical benefits without imposing any excessive financial burden on either the patients or hospitals.

## Supplementary Information

Below is the link to the electronic supplementary material.

Supplementary Material 1

## Abbreviations

CR-POPF: Clinically relevant postoperative pancreatic fistula
DGE: Delayed gastric emptying
DPC: Diagnosis procedure combination
OPD: Open pancreaticoduodenectomy
PD: Pancreaticoduodenectomy
POD: Postoperative day
POPF: Postoperative pancreatic fistula
RPD: Robotic pancreaticoduodenectomy
