# Supplementary material for: Evaluating the value of robotic versus open pancreaticoduodenectomy: improved clinical outcomes without compromising hospital revenue per admission
Source: Surg Today. 2026 Aug 6;56(11):2992–9. doi: 10.1007/s00595-026-03451-5 (PMC13722742; doi:10.1007/s00595-026-03451-5)
Supplement: Supplementary file 1 [file 595_2026_3451_MOESM1_ESM.docx]

| **Supplementary Table 1. Economic Outcomes Between Robotic and Open Pancreaticoduodenectomy,**  **Including Estimated Intraoperative Surgeon Labor Costs and Depreciation Expenses for Robotic System** | | | |
| --- | --- | --- | --- |
| Characteristics | RPD group **(n** = 41**)** | OPD group **(n** = 42**)** | P value |
| Estimated Intraoperative surgeon labor costs, Mean ± SD |  |  | 0.176 |
| Yen | 129,668 ± 34,745 | 144,187 ± 45,892 |  |
| USD* | 926 ± 248 | 1,029 ± 327 |  |
| Hospital profit, **m**ean ± SD |  |  | 0.306 |
| Yen | 2,058,411 ± 328,057 | 2,228,639 ± 567,249 |  |
| USD* | 14,702 ± 2,343 | 15,918 ± 4,051 |  |
| Hospital operating margin A, median (IQR) | 0.71 (0.62–0.70) | 0.80 (0.78–0.83) | < 0.001 |
| Abbreviations: RPD, robotic pancreaticoduodenectomy; OPD, open pancreaticoduodenectomy; SD, standard deviation; IQR, interquartile range. *Calculated based on an exchange rate of 140 yen per U.S. dollar (average from 2022 to 2024). | | | |
